# Managing under austerity: a qualitative study of management–union relations during attempts to cut labour costs in three South African public hospitals

**DOI:** 10.1108/JHOM-11-2022-0324

**Published:** 2024-03-05

**Authors:** Thanduxolo Elford Fana, Jane Goudge

**Affiliations:** Faculty of Health Sciences, Centre for Health Policy, School of Public Health, University of the Witwatersrand, Johannesburg, South Africa

**Keywords:** South Africa, Trade unions, Management, Hospital, Labour relations

## Abstract

**Purpose:**

In this paper, the authors examine the strategies used to reduce labour costs in three public hospitals in South Africa, which were effective and why. In the democratic era, after the revelations of large-scale corruption, the authors ask whether their case studies provide lessons for how public service institutions might re-make themselves, under circumstances of austerity.

**Design/methodology/approach:**

A comparative qualitative case study approach, collecting data using a combination of interviews with managers, focus group discussions and interviews with shop stewards and staff was used.

**Findings:**

Management in two hospitals relied on their financial power, divisions between unions and employees' loyalty. They lacked the insight to manage different actors, and their efforts to outsource services and draw on the Extended Public Works Program failed. They failed to support staff when working beyond their scope of practice, reducing employees' willingness to take on extra responsibilities. In the remaining hospital, while previous management had been removed due to protests by the unions, the new CEO provided stability and union–management relations were collaborative. Her legitimate power enabled unions and management to agree on appropriate cost cutting strategies.

**Originality/value:**

Finding an appropriate balance between the new reality of reduced financial resources and the needs of staff and patients, requires competent unions and management, transparency and trust to develop legitimate power; managing in an authoritarian manner, without legitimate power, reduces organisational capacity. Ensuring a fair and orderly process to replace ineffective management is key, while South Africa grows cohorts of competent managers and builds managerial experience.

## Introduction

The 2008 global financial crisis led to a period of austerity in many countries. The negative or slow economic growth in low- and middle-income countries (LMICs) resulted in tax revenue shortfalls, exacerbated by diminishing foreign financial assistance. Health spending as a percentage of gross domestic product (GDP) declined on average by 3% in 128 LMIC's from 2008 to 2010 (Overmans and Noordegraaf, 2014; Massuda *et al*., 2018; Maley, 2019). Numerous governments instituted austerity programs to address increasing deficits and unsustainable debts (Kerasidou *et al*., 2016; Blecher *et al*., 2017), recently increased by the COVID pandemic. The 2008 crisis had wide-spread ramifications for the size and structure of the public sector, as health, education and social and welfare services were restructured and cuts in labour costs were implemented through pay restraint, job losses and outsourcing (Lazes *et al*., 2012; Johnson *et al*., 2019). Employees experienced reductions in pay or benefits and development opportunities, as well as job insecurity, work overload, demotivation and well-being issues (Pepple and Olowookere, 2020).

In LMICs the public sector is often highly unionised. An organisation is a ‘field of power relations’ (Shiffman, 2015) with different actors (unions, management and employees) drawing their power from different sources and using various tactics to further their interests (Johnson *et al*., 2019). Cutting labour costs, not a neutral exercise, involves power struggles between the different actors. In periods of austerity, management and unions are faced with a challenging task of striking a balance between the need to adapt to the new constrained circumstances and the needs of the employees; failure to strike an accepted balance can result in either management, or unions, losing their legitimacy (Glassner *et al*., 2011).

In this paper we examine management–unions and union-employee relations in times of austerity in three hospitals in a poor rural province in South Africa. We look at strategies used by management to reduce labour costs, the power struggles that ensued, the underlying sources of power and the way they were expressed. The study also sought to establish which hospitals were able to strike the balance described above, and why their strategies were successful. Given the recent COVID-19 pandemic, austerity is likely to affect health systems in many countries over the next few years, and it is crucial to understand how its impact might play out.

### Bourdieu's field theory

There are different forms of field theory (social psychology (Lewin, 1951); organisational and institutional studies (DiMaggio and Powell, 1983; Bourdieu, 1985), and it is a useful general explanatory approach for the social sciences (Martin, 2003). In this paper we focus on Bourdieu's work, who argues that a field is a social typology with different interconnected domains or sources of power (or capital) that actors use to secure particular outcomes: **social capital** (networks of connections, such as shop stewards enlisting union members to oppose a management strategy); **financial capital** (command over economic resources, such as management not appointing replacement staff); **cultural or ideological capital** (enlisting the support of union members based on an understanding of common discourse, such as the negative effects of casualization of labour) and **symbolic capital** (resources available to an individual on the basis of honour, prestige, recognition or position. For example, individuals may hold more leverage in social or political arenas based on their prior experiences (e.g. veterans from the struggle against apartheid or their position as a manager.) (Bourdieu, 1985). **Symbolic power or violence** is the tacit, almost unconscious discipline used against another to confirm that individual's placement in the social hierarchy, at times in individual relations but often through system institutions (Bourdieu and Wacquant, 1992), for example education (the use of the Bantu education system during Apartheid is an extreme example), or the power that managers have to impose certain working conditions.

Every field is “based on a working consensus as to the nature of the game, and people take predictable sides due to the more general structuring of social space” (Bourdieu, 1985; Martin, 2003) (p. 23). Yet this coherence is a dynamic one, for “every field is the site of a more or less overt struggle over the definition of the legitimate principles of the division of the field” (Bourdieu, 1985) (p. 734). What is at stake is not just who the immediate winner is, but what type of actors will dominate the field in the future. An organisation's ability to achieve the balance described above (balancing the needs of employees and patients while adapting to the new constraints) depends on how the various sources of power are used, the outcome of the power struggles and whether and how rules of the game are changing as a result (Martin, 2003).

### Background

Between 1994 and 2013, the new democratic government in South Africa enabled sustained economic growth and was able to protect social-sector spending, even during the 2008 global financial crisis, due to it counter-cyclical fiscal stance. However by 2013 the global financial crisis had a negative effect, economic growth slowed and there has been a decline in real terms health budgets from 2013 to 2020 (Blecher *et al*., 2017). In this environment, higher-than-inflation increases in wages and the costs of medical products, proliferation of medical negligence litigations (Maphumulo and Bhengu, 2019) and internal inefficiencies, corruption and wasteful expenditure have led to the need for a period of austerity (Pillay and Mantzaris, 2017). Numerous cost-saving exercises have been implemented, included the freezing of posts, outsourcing services, prioritisation of core items over the less essential, reducing infrastructure spending and investment in staff training and development (Malelelo-Ndou *et al*., 2019). The particular province studied centralised decision making, introducing a provincial cost control committee that made every expenditure decision (Wishnia and Goudge, 2020).

In South Africa, trade unions have played a major role in the transformation of the country; in the late 1980s, they formed the principal vehicle for internal opposition to the apartheid regime. In 1990s the Congress of South African Trade Unions (COSATU) formed an alliance with the African National Congress (ANC). As a result, unions had a voice in ANC policy fora and the right to nominate a proportion of ANC parliamentary candidates. COSATU's links to government drove changes to labour legislation which, in turn, have strengthened the rights of unions and of individual employees since democracy (Dibben *et al*., 2012). Public sector unions' involvement with government has also enabled them to consistently obtain above inflation salary increases through centralised bargaining. Despite the existence of institutions responsible for managing collective bargaining with public sector unions (such as National Economic Development and Labour Council), strikes have on occasions been violent, with little regard for patient care, or the safety of staff who chose to continue working (McQuoid-Mason, 2018), and at time have led to the loss of life (such as the Marikana massacre (Chinguno, 2013).

Bourdieu's theories have relevance to South Africa particularly in explaining the on-going violence seen both in the workplace and local politics (Burawoy and Von Holdt, 2012). Writing in 2012 von Holdt argues that while the symbolic order of apartheid was dismantled and replaced by the institutions of democracy (a constitution, an elected parliament, independent judiciary and media), there is considerable conflict over the meaning of skill (of which the white minority claimed monopoly under apartheid) and so whether qualification or allegiance is most important in making civil service appointments, and in turn to conflict over authority, both managerial and political. Workers often challenge, sometimes using violence, management in the workplace (Baloyi, 2019), communities riot to protest at the failure of elected leaders (Chiguvare, 2022; Lali, 2022; Njilo, 2022) and political leadership is contested such that assassination is increasing (Ellis *et al*., 2022). Von Holt describes local government mayors removed through violent protests, resulting in poorer governance, and institutional instability due to repeated change in leadership (Burawoy and Von Holdt, 2012). Baloyi describes the instance of a clinic manager too scared to go to work due to intimidation from her staff (Baloyi, 2019). Von Holt's point is that the transition to the democracy has not led to new consensus, or new order, through symbolic power. The rules of the game, and who is in positions of authority, are contested.

A decade later, the appointment of managers without the necessary skills has led to the near collapse of key state institutions (health services (Stevenson, 2022); paediatric (Metelerkamp, 2022) and cancer services (Ho, 2022) as well as, for example electricity provision, the national airline and tax revenue collection services). This has been exacerbated by state capture (through the appointment of people without appropriate qualifications in order to threaten them with exposure and so enable the control of key government services and the associated funds) (Ashton, 2021). There has been a detailed public documentation of state capture by the Zondo Commission of Inquiry (Government of the Republic of South Africa, 2022), and although it is unclear whether the judicial system has sufficient capacity to bring those guilty to book, there is a recognition that things need to be done differently (Ashton, 2021; Mcebisi, 2022). A key question is whether public service institutions can, or are, re-making themselves, under circumstances of austerity, in a way that matches the ideals of the democratic era.

## Methods

### Study design

We used qualitative methods with a case study design, to gain an understanding of the strategies used in the three public hospitals to cut labour costs and their impact in times of austerity. The use of qualitative case studies enabled us to understand the sequence of events, relationships between the actors involved, the history of their engagement, motivation, reasons for use of the various strategies and responses to the strategies of others.

### Study sites

This study was conducted in three public hospitals in a large rural province in South Africa, with high levels of unemployment and poverty. A combination of purposive and convenient sampling strategies was used to select three facilities. First author (TF) had worked for several years in the province, and knew two of the hospitals well, and so was able to gain access to staff for interviews; the third had a reputation for good performance, and so was chosen to identify what worked.

### Selection of participants and data collection methods

We conducted interviews (*n* = 47) with managers, shop stewards and employees, as well as focus group discussions (*n* = 12) with employees (*n* = 84) from April to September 2019 (Table 1) (Fana and Goudge, 2021). The participants were selected through purposive and convenience sampling. Manager and shop stewards were selected purposively because they were responsible for management, supervision of staff and day-to-day decision making and running of the hospital. Managers interviewed included Chief Executive Officer, Chief Medical Officer, Clinical Manager, Deputy Director Human Resources, Assistant Director Human Resources Management, Patient Administration, Operations, Finance, Nursing Manager, Area Manager and Operation Managers Nursing in each hospital. Convenient sampling was used to select employees, based on who was available and willing to participate in the study.

Focus group discussions were held with shop stewards, clinical and non-clinical staff. These were supplemented with an in-depth interview with one person from each group to counter the group effect. Participants were asked to introduce themselves (position, education and experience), and to describe their experiences of working in the hospital, and management unions and employee relations (within, between sections and different actors). Participants were asked about changes they have observed or experienced and how they were communicated, and by whom, procedures and processes for raising issues, and reasons for them, how issues were dealt with and by who. The facilitator explained to participants that due the nature of focus groups, confidentiality of what was said could not be guaranteed. The interviews and focus group discussions were audio recorded and saved in a password-protected computer. Policy on management of public hospitals, performance management and development, training and development, disciplinary procedure, recruitment and selection circulars and policies, hospital organograms, minutes of top management and Institutional Transformation Unit meetings were reviewed.

The researchers amended the questions during data collection to take into consideration knowledge already gained from the previous interviews and focus group discussion. Data was discussed on an ongoing basis and when no new information was emerging and data saturation was reached, data collection was stopped.

### Data analysis

TF wrote field observation notes after each interview. Two field workers transcribed data, and TF checked the transcriptions for accuracy. TF extracted data from the interview transcriptions into a single document for each facility, either as quotes or providing summarised version of events. We (TF and JG) re-read the data and, using an inductive approach, we identified common and divergent themes across the multiple data sources, and across the hospitals, identifying differences and similarities. Each author conducted this process independently, we then compared our results, and differences were resolved through discussion, ensuring dependability of our findings (Lincoln and Guba, 1986). The agreed high-level themes were management stability and leadership style, unions and their leadership, strategies to reduce labour cost (outsourcing, bringing in free, cheaper and inexperienced staff, and getting greater productivity from existing staff) and the power struggles that ensued.

We then identified Bourdieu's framework as a useful way to frame the sources of capital/power that were used by the actors. TF then re-coded the data according to the different sources of power (capital): social, financial, cultural or ideological capital and symbolic capital. We jointly interrogated the data under each element of the framework, discussing the strength of the evidence including pieces of evidence were divergent, to ensure none of the divergent evidence was lost in the process of developing a narrative. In preparation of the article, we continued to visit both the raw and summarised data to confirm descriptions of the content, enrich descriptions of the context, provide clarity and check lines of argument that emerged. This iterative process helped to ensure the credibility, dependability and confirmability of our findings (Lincoln and Guba, 1986).

Field notes were used to inform our understanding, but we do not use verbatim quotes from the notes. However, the field notes did enable TF to be more aware of any biases, helping to ensure confirmability and to develop deeper insights while in the field. No analysis software was used in the analysis process, only Microsoft Word.

### Positionality

TF worked for 10 years as a human resources practitioner at one of the facilities, during which time he interacted with staff from other hospitals in the district and in province. The researcher spent 7 of the 10 years as a National Education Health and Allied Workers Union (NEHAWU) shop steward and the hospital's branch secretary. In this capacity, the researcher was involved in negotiating with management on behalf of the employees, mediating disputes and grievances between departmental staff and employees and representing employees at disciplinary hearing. TF's previous role may have affected some participant's willingness to participate, as four managers (Labour Relations, Administration, Area Manager Nursing and Social Worker) out of 33 managers across the 3 hospitals refused to participate. Three groups of staff (food service aids, laundry and cleaners) in 1 of the hospitals also refused to participate as they did not have permission to participate from the section manager despite several attempts to explain the purpose of the study. However other groups of staff at similar level (porters, mortuary attendants) did participate, and shop stewards, who represent a wide range of staff, were well represented in the study. Others were excited about participating as the study gave them the opportunity to share some of the challenges in times of austerity.

### Patient and public involvement

Hospital managers contributed to the research focus in the planning stages, however, no patients or members of the public were involved in the research design, analysis nor dissemination of the findings.

### Ethical approval

The University of the Witwatersrand's Human Research Ethical Committee (Medical) and the Provincial Department of Health, Health Research Committee, granted the ethical clearance for this study (clearance certificates number M181143 and 201903–008). Permission was obtained from the district and the head of the institutions. Informed consent was obtained prior to data collection. We protected the confidentiality of participants by: a) using a numbering system to name data files, securely storing the document linking names and numbers separately; b) removing identifying information from all transcripts and quotes and c) referring to participants position in the hospital in very general terms (e.g. manager).

This work was supported by the National Research Foundation through the SARChI chair for Health Systems and Policy Research at the Centre for Health Policy at the School of Public Health, University of the Witwatersrand.

## Findings

### The hospitals, management and unions

All three hospitals are run and funded by government. Hospital A is a specialised provincial hospital, with less than 200 staff members. Hospital B is a tertiary hospital, serving a poor community with high levels of unemployment. It has more than 2000 staff members. Hospital C is a general hospital with an unclear status (regional or district), located in a relatively wealthy community, that used to serve mainly white and coloured community prior to democracy. It has more than 600 staff members.

In Hospital A, there had been three CEOs and three nursing service managers within a period of five years. The union-management meetings were no longer held as scheduled and sometimes unions were only informed on the day that a meeting would be taking place. One union had decided not to engage with the current CEO anymore. In 2016 in Hospital B, union strike action demanding the removal of the old CEO whom the unions claimed was underperforming, led to the appointment of a new CEO and a new Director of Human Resources. The new management established democratic management practices: “*This CEO consults and involves unions in planning and when things do not go as planned, the CEO comes to explain; this CEO knows how to work with unions.*” (FGD: Shop steward Hospital B). In Hospital C, only the Chief Medical Officer and Finance Manager, among the top management, had been employed at the hospital for less than four years. However, labour relations were not great; union and management meetings were often not held as per the schedule, and some managers did not attend management meetings as they feel that their inputs were not valued.

The majority of the staff in the three hospitals belonged to three major unions: the Democratic Nurses Organisation of South Africa (DENOSA) representing nurses; the National Education, Health and Allied Workers Union (NEHAWU) and Health and Other Services Personnel Trade Union of South Africa (HOSPERSA) whose members were staff across all grades (porters, cleaners, kitchen staff, laundry, maintenance, mortuary attendants, nurses, administrators, allied healthcare workers, managers, etc.). Newer unions have emerged, due to dissatisfaction with the established unions, and some employees belonged to Public Servants Association of South Africa (PSA), Public and Allied Workers Union of South Africa (PAWUSA) and South African Liberated Public Service Union (SALIPSU/SAPSU).

To participate in discussions with management, unions had to have sufficient membership, and so unions competed for members. The provincial policy of not replacing non-clinical staff who left, and the attempts by hospital management to outsource certain functions, affected the distribution of membership and so created conflict between unions. The unions with experienced and knowledgeable shop stewards, who effectively represent their members, were gaining membership; in all the three hospitals there were employees who had dual trade union membership.

In Hospital A, many long serving, experienced shop stewards had retired. The new shop stewards occupied low operational level positions and had not attended any training: *“Members are not satisfied with us because we are like them, we know nothing.”* (FGD: Shop Stewards Hospital A). One employee stated “*I am no longer reporting issues to shop stewards. They won't do anything. The shop steward that is knowledgeable does not attend to the complaint, as he is a friend of my supervisor”* (FGD: Administration Hospital A). In Hospital B, the majority of the unions had managed to retain some experienced shop stewards: *“we are fortunate in that some of our shop stewards are serving in the provincial level, and that gives us access to greater information*.*”* (Manager Hospital B). In Hospital C, experienced union leadership had left due to retirement, and the younger staff were less interested in union activities: *“It is unfortunate that NEHAWU is no longer what it used to be. Shop stewards are no longer serving the interest of the workers but are only interested on benefiting themselves”* (FGD: Shop Sewards Hospital C).

### Attempts to reduce labour costs and the resulting power struggles 

#### Outsourcing

Outsourcing was a key strategy for reducing labour costs.

1. Outsourcing laundry services in hospital A

In Hospital A, management proposed to outsource the laundry services and made plans for the remaining laundry staff to be moved to the other departments. NEHAWU rejected the proposal (as it affected their members), and in a follow up meeting, in the absence of NEHAWU, DENOSA and HOSPERSA agreed to management's proposal. NEHAWU challenged the management's decision, highlighting that management had recently bought new machinery and renovated the laundry. The provincial head office supported NEHAWU, and the outsourcing contract was cancelled after 3 months.

A NEHAWU shop steward explained the union's position: “*Our union federation COSATU is against privatisation and casualization of labour; we were against management proposal right from the beginning”* (Shop steward Hospital A). A general assistant expressed this view in more personal terms: “*we felt that our jobs were under threat. We told the shop stewards we didn't want it”* (FGD: General Assistants Hospital A). Previous experiences had shaped employees' views: “*In the past, when the hospital used private cleaning companies, most of the people working in that company were related to people in authority in the hospital. Few months later those people were employed here. We do not trust these people*.” (FGD: Cleaners Hospital A). The episode also led considerable tension between unions.

- Outsourcing artisanal skills in hospital B

In Hospital B, management proposed to outsource artisanal type tasks (e.g. welding, electrification, maintenance) as many employees had retired or left. Management explained that replacing non-clinical staff was prohibited due to austerity, and that outsourcing these particular services allowed them to be paid for under the goods and services budget (rather than under the salaries budget), while preventing costly breakages in hospital equipment and machinery. Management assured the affected staff that their jobs were safe. The management proposal was accepted by both unions and the employees.

A senior manager explained their strategy: *“We are always making sure that the unions are on board*. *When things do not go according to plan, we go back and explain.”* (Manager Hospital B). One of the remaining artisanal workers explained that he trusted management: “*We are happy, we know our jobs are safe. We were consulted by management and unions about the plan.* (FGD: Junior Staff Hospital B).

- Using existing staff with artisanal skills in hospital C.

In Hospital C, general assistants (e.g. cleaners) with artisanal skills were asked to carry out welding, plumbing and electrification tasks. These arrangements were between the employee and their immediate supervisor; unions were not involved. Over time, the employees started complaining about having to do jobs at a higher grade than their current positions, without any compensation, or guarantee that they will get the positions should they be advertised. Management responded that none of the employees had an official acting letter, and that filling of non-clinical positions was prohibited due to austerity.

Some staff accused management of filling management positions and not doing enough to fill those at lower levels. Another participant said: “*I am happy, this is an opportunity to learn, practice and develop myself for future opportunities elsewhere*. (FGD: Junior Staff Hospital C). Another participant explained the dilemma they faced: “*If we do not do it, our hospital and community will suffer. It is unfair on us, but we are doing it, to ensure that services are continuing with minimum disruptions in the hospital* (FGD: Shop Stewards Hospital C). Senior management acknowledged the negative impact on union-management relations. Union-member relations were also strained as employees didn't believe unions were making enough effort to pressurise management; some switched unions, resulting in further tensions between the unions.

#### Bringing in free, or cheaper, inexperienced staff

1. Finance interns in hospital B

In Hospital B, management proposed that interns be brought in to assist in the finance section. Finance posts had been used to appoint staff in other departments, and understanding this, unions agreed to the proposal. A manager explained the impact: “*The backlogs have been cleared, and orders and payment are done within specified time frame of 21 days. There is a decline in complaints for non-payment; things are moving now”* (Manager Hospital B).

- The Extended Public Works Program in hospital C

In Hospital C, management proposed seeking assistance from the Department of Public Works, Extended Public Works Program (EPWP) for forty general assistants to assist with cleaning. The EPWP, run by the Department of Public Works, employs people from the local area, who would otherwise be unemployed, to work on public sector projects; the extra labour would be free of charge to the hospital. Management explained to the unions that the use of EPWP general assistants was only a temporary measure. DENORSA and HOSPERSA supported the management's proposal, but NEHAWU and PSA rejected it. Management went ahead, but NEHAWU members demonstrated (toyi-toyed) and chased the EPWP workers away from the hospital.

One general assistant explained: “*We knew right from the beginning that they were going to continue and implement their plan irrespective of what we say, as they did with laundry. We were not going to let it slide like we did the last time and hence we toyi-toyed.”* (FGD: General Assistants Hospital C).

A manager didn't understand the response of the unions: “*I do not understand*. *We are short staffed, and this was an opportunity to alleviate workload and pressure from the permanent staff*.*”* (Senior manager Hospital C). Another manager expressed mistrust: “*I do know what the unions told the workers, for them to behave in that manner. I still have to find time and talk to the workers myself.*” (Manager Hospital C). It seems this manager hadn't prioritized communication with unions or staff.

The episode led to further tension between unions. DENOSA was unhappy that their members had to continue assisting with the cleaning, and NEHAWU accused DENOSA of supporting privatisation and outsourcing initiatives, to the detriment of their fellow workers.

#### Obtaining greater productivity from existing staff

1. Incapacity leave

Incapacity leave is granted when an employee is sick and they have used their normal 36 days of sick leave in a three-year cycle. An Ill-Health Committee should review the applications for incapacity leave and provide recommendations to management. In Hospital B the committee was effective and fully functional. In both Hospital A and C, the committee had not been functioning for some years, and there were employees who had been on incapacity leave for 5 years. When employees raised concerns over those who were at home and on full pay, management revived the Ill-Health Committee.

Some of the staff were requested to report back to work, while others were recommended for ill health retirement and some of their posts filled with new employees. One participant explained how management and unions collaborated: “*Unions played a very crucial role; they cooperated with management throughout the process and there were no appeals to the decisions taken” (*Manager Hospital C).

- Asking staff to work beyond their scope of practice

In all the three hospitals, staff were expected to work outside of their professional scope of practice. Enrolled nurse managed wards and administered treatment without supervision. In Hospital A and C, nurses complied with these requests due to fear being reprimanded. One complained that they were not allowed to mention tasks above their scope of practice in their performance evaluations as it might lead to an audit query. When the staff member makes a mistake: “*you will be on your own, the managers are not willing to defend and support you when you face disciplinary hearings, even though you were assisting the patients”* (FGD: Enrolled Nursing Assistants Hospital C).

In Hospital B, in contrast, participants had been consulted on how to manage the workload: “*you are doing it because you were asked to and not forced, although you will wake up with painful bones [due to tiredness].*” (FGD: Enrolled Nursing Assistants Hospital B). Participants described the benefit of doing higher level tasks: *“you learn and acquire new skills set, especially if it is within your field of work. When you complete a task successfully you feel proud, satisfied and that drives and motivates you as an employee”* (FGD: Enrolled Nursing Assistants Hospital B). Mistakes did happen and staff had to attend disciplinary hearings, however: *“We are fortunate, we do get the support of our manager. That makes it easy for us to help out when the manager asks”* (FGD: Enrolled Nurses Hospital B).

- Installing cameras

In Hospital A, management proposed to install security cameras. NEHAWU voiced objections, stating there were already security guards, and so it was a waste of money. Both DENOSA and HOSPERSA agreed with management's proposal. Management went ahead and installed the cameras, starting at the kitchen. When kitchen employees started to ask questions, their concerns were not taken seriously by other staff or the unions. Days later, when installations began in the wards and nursing stations, DENOSA objected, saying the cameras were a violation of patients and staff privacy. NEHAWU refused to support DENOSA and HOSPERSA, and in the end no union challenged the management's actions.

Staff expressed their resentment, blaming the unions: *“I do not know why they have agreed to this nonsense in the first place. How can we work like this? Are we paying union fees for this nonsense” (FGD: Nurses in Hospital A).* However, many nurses were caught sleeping on duty, and managers were able to obtain evidence when staff regularly arrived late. Staff were charged with misconduct and given warning letters.

## Discussion

In this paper we use Bourdieu's field theory to understand the interaction between the actors in public hospitals (employees, unions and management) in their quest to provide health care and minimise disruptions, while cutting labour costs. We have shown that the hospitals had three broad strategies: outsourcing, bringing in cheaper inexperienced staff and getting greater productivity from existing staff. Table 2 provides a summary of these strategies, the source of power and the outcomes.

In Hospital B, protests by unions had led to the removal of the previous CEO. Because the new management had chosen a participative leadership style, and shop stewards were experienced, union-management relations at the time of the research were open, collaborative and based on mutual respect and trust. Despite cuts in training budgets, management ensured that in-house joint union management training on labour relations continued, and this provided an opportunity for discussion of any tensions. Their cost cutting strategies (outsourcing artisanal tasks, bringing in interns, asking nurses practicing out of scope) were successful because there was trust, and the engagement processes generated legitimate (symbolic) power on which to draw.

In Hospital A and C, management appeared to be less skilled and there was turnover among the union leadership. Management made use of financial and cultural power (by refusing to pay employees for additional work, relying on staff feeling guilty about letting down their colleagues and community despite not being rewarded for working extra). Management also took advantage of the divisions that existed between unions (over outsourcing, installation of cameras and use of EPWP employees), and used their symbolic power (to instruct staff to do things beyond their scope of practice). They failed to defend their staff working beyond their scope of practice, reducing employees' willingness to take on extra responsibilities. With little pre-existing trust, or on-going collaboration, management–union engagement didn't generate any legitimate power. As a result, the unions used their ideological and social power to protest against the use of EPWP employees, and to reject casualization or outsourcing of laundry services.

Shiffman used Bourdieu's theory of field of power relations to examine power in the global health arena (Shiffman, 2015). In the global health arena, there is no one organisation that makes final decisions, and there is insufficient attention to legitimacy, due in part to an erroneous belief held by many global health actors, that expertise and moral authority are the preserve of educated elites, and that their intended beneficiaries have little knowledge or capacity to analyse their own realities (Shiffman, 2015). In a hospital setting, there are leaders with responsibility for taking decisions, even if they are constrained by available funding and decisions made higher in bureaucracy. However, there is often insufficient attention to the generating legitimate power, with some public hospital managers, often those with insufficient skill and insight, assuming that their position (and associated power) means they don't need to take the time to explain the constraints and to seek consensus on how to move forward, leading to the failure to earn the respect of the employees.

In the instance of Hospital B, the ousting of the previous leadership had had a positive outcome, but this might not have happened. For public institutions to function well, there needs to be a process that allows for renewal and replacement of leadership without instability, without union protests. In South Africa, this should involve reviews of hospital managers' performance by provincial officials, and their replacement if necessary. That unions protests led to the removal CEOs suggests that the ‘rules of the game’ do not necessarily result in a fair process, which: a) may lead managers to be subject to unfair workplace processes with respect to their own employment, which in turn won't encourage others to take on managerial responsibility, and b) is likely to lead to instability and organisational decline, if unions have sufficient power to remove a manager who has different perspective. In the post-state capture era, some institutions have been stabilised (Van Heerden, 2022), but others have not. Unfortunately some provincial health administrations in South Africa are poorly functioning and so are unable to steer process for appropriate processes for the renewal of managers (Stevenson, 2022).

In many high-income countries, decentralisation of bargaining processes to the organisational level (Stanton, 2002), payment of hospitals through case mix formula (Stanton, 2000), marketisation (Greer *et al*., 2013), has raised concerns about the diminishing role of unions in the health sector. However, evidence of the effects has been varied (Schwarz and Koziara, 1992), with continued union activity in some settings (Lloyd, 1997), and de-intensification of work in others (Willis *et al*., 2008). The UK, due to the chronic under funding of the NHS compounded by the COVID pandemic, has seen a recent series of strike action by nurses and doctors, despite considerable decentralisation and marketisation of the NHS (Iacobucci, 2023).

Many low- and middle-income countries don't have capacity within their Ministries of Health to manage labour relations. For example, a WHO assessment of public sector institutional capacity for health workforce governance found that India, Indonesia, Bangladesh do not have capacity, but Thailand does. Industrial action frequently disrupts health care in Nigeria, the cause often conflict between different professional groups as much as renumeration (Suleiman and Stewart, 2020). In Iran, industrial action has been prompted by a range of problems (poor training, unclear job boundaries and unpleasant working environments) (Mahsa *et al*., 2023). In China, due to authoritarian management and compliant trade unions, conflict can't be managed through collective channels, but is often channelled through informal, individual activities (Cao, 2014). In South Africa, after a series of strikes and the collapse of mediation, a constitutional court ruling decided that a recent multi-year public wage agreement did not need to be upheld as the government had insufficient funds to meet the agreement (Smit, 2022).

Training and development of labour relations can build resilience (Maley, 2019; Maphumulo and Bhengu, 2019), minimise the impact of austerity and help in dealing with uncertainty, as well as to improve performance, morale and employee satisfaction (Overmans and Noordegraaf, 2014). Unions require well equipped and capacitated staff, who can construct an agenda for workplace change, communicate it effectively to members, negotiate and liaise with employers and remain open and responsive to diverse worker needs (Pyman *et al*., 2010; Lazes *et al*., 2012; Bailey and Robertson, 2015; Bischoff *et al*., 2018). For organisations to capture the value that unions can add, their leaders must have sufficient skill and insight to move away from autocratic management and adversarial industrial relations to a cooperative partnership (Wood and Glaister, 2008; Hjalager *et al*., 2009; Pyman *et al*., 2010; Schmidt *et al*., 2019). These are the necessary skills, and the rules of the game that need to be agreed upon, to enable public institutions to re-build themselves in a way that matches the ideals of the democratic era.

### Implications for management practice

In the South African context, given the history of apartheid, labour relations are particularly complex, with skill and authority regularly being contested, often in violent ways. Training on labour relations for employees, union staff and management is important for strengthening public service organisations in any country, and it is particularly important: a) in the South African context because of contestation of skill and authority, and b) during times of austerity when tensions increase.

With the contestation over whether it should be allegiance or skill that determines appointments, managers may not have the necessary skills, and so there is need to have adequate formal and fair processes to remove those managers who fail to perform adequately.

### Strength and limitations

Our study extends the existing research on austerity and employment relations, looking at strategies to reduce labour costs, the power struggles that ensued in frontline public health organisations. Another major strength of our study is the variety of employee and managers roles among the participants. The hospitals were selected based on prior knowledge, ease of entry and access. As a result, although the study hospitals are typical of many South African public hospitals, the findings cannot be generalised to all public hospitals. Moreover, the institutions are located in urban areas and are better resourced than those in rural areas. The use of convenience sampling may have prevented some employees participating, as those busy working or on leave may have been excluded. The participation of shop stewards who represent diverse employee groups provided a range of views even though three groups of staff from one of the three hospitals did not participate in the study.

## Conclusion

Our findings highlight the importance of an organisation's ability to find a balance between the new reality of reduced financial resources and the needs of staff and patients. Finding an appropriate balance requires competent unions and management, promoting consultation, transparency and building trust to develop legitimate power; failure to do so weakens the organisation's ability to ensure continued provision of healthcare services. Not only are the rules of the game uncertain, but conflicts reduce organisational functioning and resilience. Building the capacity to ensure a fair and orderly process to replace ineffective management is key, while South Africa grows cohorts of competent managers and builds managerial experience.

## Figures and Tables

**Table 1:** Demographic profile of study participants

Interviewees	Hospital A			Hospital B			Hospital C		
	Race	Gender	Experience (years)	Race	Gender	Experience (years)	Race	Gender	Experience (years)
Senior management	B	F	35	B	M	10	C	F	30
	–	–	–	W	M	15	–	–	–
	C	M	10	B	F	25	W	F	35
	–	–	–	B	M	10	–	–	–
	–	–	–	B	M	15	–	–	–
	B	F	5	B	F	10	B	M	5
	–	–	–	B	M	4	–	–	–
Middle management	B	M	7	–	–	–	W	M	30
	B	M	5	B	M	10	B	M	40
	B	F	20	–	–	–	B	F	5
	C	M	8	C	M	15	C	M	10
Shop Stewards	B	M	7	B	F	10	B	F	3
	W	F	1	C	M	2	C	M	2
	B	M	3	B	F	15	B	F	15
	–	–	–	–	–	–	B	M	2
	–	–	–	–	–	–	C	F	5
	–	–	–	C	M	10	–	–	–
Total Interviews	10			13			12		
Focus group participants									
Nurses	3 B 2 B 2 C	F M F	5, 9 and 20 6 and 10 7 and 10	3 B 2 B 3 W 2 C	F M F F	3, 8, 16 9 and 20 7, 9, 11 11 and 13	2 B 2C 1 C 2 W	F F M F	7 and 11 15 and 23 11 15 and 21
Allied Healthcare Professionals	2 B	F	15 and 10	2 B	M	12 and 13	2 B	M	7 and 8
	2 W	F	15 and 3	2 W	F	8 and 18	1 I	Ms	11
	2 C	M	10 and 4	3 C	F	3, 8, 11	3 W	F	7, 9, 12
General and Administration	2 B	M	35 and 10	3B	F	7, 9, 16	2 B	F	11 and 15
	3 B	F	10, 9 and 7	3W	M	70.8, 20	2 C	M	12 and 14
	1 I	F	10	2 C	M	11 and 17	2 C	F	8 and 18
Shop stewards	2 B 2 B 1 W 1 C	M F F M	7 and 3 1 1 4	3 B 2 B 2 C 1 W	F Ms M M	1,10,15 10 and13 2 and5 1	2 B 1 B 2C 1 C 1W	F M F M M	3 and 15 2 5 and 8 2 7

**Note(s):** M = male; F=Female; B=Black; C=Coloured; W=White; I=Indian

**Table 2:** Strategies to cut labour costs, outcomes and sources of power

Strategy		Hospital A	Hospital B	Hospital C
*Outsourcing*		*Outsourcing laundry services*	*Outsourcing artisanal/maintenance tasks*	*Using existing staff for artisanal/maintenance tasks*
	Outcome	Laundry outsourcing contract cancelled; tension between unions and between unions and management	Outsourced artisanal tasks; equipment maintained; Workload more manageable	Some staff did extra work; dissatisfaction about not being compensated, but equipment maintained
	Source of power	*Management:* Division between unions	*Management:* Legitimate/participatory management processes	*Management:* Financial – refused to pay for additional work; Cultural – staff felt responsibility towards hospital community
		*Unions:* Higher government body	*Unions:* None used/agreed with strategy	*Unions:* None
*Free or cheaper labour*			*Finance interns*	*Extended public works program (EPWP)*
	Outcome		Interns joined finance department; decrease in workload; satisfied staff; backlog cleared	EPWP workers chased away; continued shortage off staff; further tensions
	Source of power		*Management:* Participatory management processes–legitimate power *Unions:* None used/agreed with strategy	*Management:* Division between unions *Unions:* Ideological power- Against casualization of labour force Social power–mobilized union members to protest
*Greater productivity from existing staffs*		*Asking staff to practice out of scope*	*Asking staff to practice out of scope*	*Asking staff to practice out of scope*
	Outcome	Staff felt used and dissatisfied; Some staff refused, and services were disrupted	Staff felt happy and satisfied; less disruption to services	Staff felt used and dissatisfied
	Source of power	*Management:* Financial – not hiring more staff Cultural–staff felt responsibility towards hospital community; Symbolic – supervisory power *Unions:* None	*Management:* Participatory management processes–legitimate power *Unions:* None used/agreed with strategy	*Management:* Financial – not hiring more staff Cultural–staff felt responsibility towards hospital community; Symbolic – supervisory power *Unions:* None
		*Installation of cameras*		
	Outcome	Cameras installed; monitored attendance; disciplined those sleeping on duty; tension between union and management; Dissatisfaction of members with unions		
	Source of power	*Management:* division among unions *Unions:* Social network power- asked other unions to oppose management but failed		
		*Incapacity leave*		*Incapacity Leave*
	Outcome	Employees who did not qualify come back to work; some vacated positions were filled others not		Employees who did not qualify come back to work; some vacated positions were filled others not
	Source of power	*Management:* Legitimate		*Management:* Legitimate
		*Unions:* None used/agreed with strategy		*Unions:* None used/agreed with strategy
